# Emergency department visits for dog bite injuries in Missouri municipalities with and without breed-specific legislation: a propensity score-matched analysis

**DOI:** 10.3389/fpubh.2024.1354698

**Published:** 2024-04-05

**Authors:** Brett Wyker, Maya Gupta

**Affiliations:** Strategy and Research, American Society for the Prevention of Cruelty to Animals, New York, NY, United States

**Keywords:** breed-specific legislation, BSL, dog bites, pit bull, injury

## Abstract

Breed-Specific Legislation is a type of law that bans or restricts ownership of specific dog breeds. Some local governments – including over seventy municipalities in the state of Missouri – have enacted Breed-Specific Legislation to prevent injuries from dog bites. Several studies from the peer-reviewed literature have found that aggressive behavior is not associated with any particular dog breeds and, since 2018, at least a dozen municipalities in Missouri have repealed these laws. To evaluate the impact of Breed-Specific Legislation on public safety, the 2010–2015 rates of emergency department visits for dog bite-related injuries in Missouri municipalities with and without Breed-Specific Legislation were compared. Propensity-score matched negative binomial regression models were used to assess the effect of breed restrictions on injury rates while balancing the samples on population characteristics and estimates of dog ownership. After matching the sample on population, housing and dog ownership estimates, no association was found between emergency department visits for dog bite injuries and whether the municipality enacted Breed-Specific Legislation. However, the incidence rate ratio of emergency room visits for dog bite-related injuries increased by 13.8% for every 1% increase in the percentage of males aged 5 to 9 in the population (*p* < 0.01). This study has found breed discriminatory laws have not reduced the risk of emergency department visits for injury from dog bites in Missouri. There appears to be no greater risk to public safety as local governments move to repeal existing breed bans.

## Introduction

The rate of dog bite related emergency department (ED) visits in the United States appear to have declined as dog ownership rates have increased (1, 2). Over the two decades between 1990 and 2010, dog ownership trends in the United States remained constant, with approximately 39% of households owning at least one dog (1). Since 2010 dog ownership has steadily increased and it is now estimated that more than half (54%) of U.S. households own at least one dog (2). Over the same period, ED visits for dog bite related injuries dropped from an average of 112 visits per 100,000 residents between 2010 and 2015 to 107 visits per 100,000 between 2015 and 2020 (3). This trend suggests that the mere presence of dogs in a community may not be associated with the risk of being bitten by a dog. Nonetheless, in the U.S., dog bites are ranked as the 8th leading cause of nonfatal injury for children under the age of 15 (3) and children – particularly boys – aged 5 to 9 are at the greatest risk for a dog bite-related injury that results in an ED visit (4–8).

Some municipalities have sought to reduce the incidence of dog bite-related injuries by placing targeted restrictions on dog ownership through local ordinances known as Breed-Specific Legislation (BSL). In most cases, BSL prohibits the ownership of specific breeds, but may also limit ownership through restrictions placed on guardianship of certain breeds (ownership fees, muzzling, signage and insurance requirements, etc). These laws are enacted as an attempt to make communities safer by prohibiting the ownership of certain dog breeds stereotyped as more aggressive than other breeds. Yet, a significant number of studies have provided evidence that BSL is unlikely to protect against serious injury from a dog bite, as breed is not associated with the likelihood of being bitten by a dog (9–11) or the strength of a bite (12–14). Patronek et al. (15) outlined an approach similar to the Number Needed to Treat from evidence-based medicine (16) to estimate the number of dogs from a targeted breed that would need to be banned to prevent an ED visit for a dog bite-related injury. Using this approach, 6,667 dogs would need to be banned to prevent a single ED visit each year based on the current estimates of dog bite ED visits and the assumption that the breed banned accounted for 15% of dog bites. This Number Needed to be Banned would grow exponentially with each ED visit prevented, calling into question the efficacy of targeted breed restriction as a viable injury prevention strategy. In fact, studies that have assessed the effectiveness of BSL in Canada (17), Spain (18), Ireland (19), Scotland (20), The Netherlands (21) and Denmark (22) have found that serious dog bite injuries were not reduced through the enactment of BSL.

To date no research evaluating the impact of BSL on ED visits in local municipalities in the U.S. has been published. For this study, medical records from hospitals in the state of Missouri (MO) were used to determine if ED visits for dog bite injuries are higher in MO municipalities without BSL than in MO municipalities with BSL. Without randomization, a disproportionate distribution of factors associated with dog bite injuries may confound the assessment of BSL’s impact on injuries. To account for the potential influence of selection bias, Propensity Score Matching (23) was used to match each BSL municipality included in the sample to municipalities with similar population (% males 5–9 and 10–14), housing (% renter-occupied units) and dog ownership (% households with one or more than one dog) characteristics.

Municipal codes in over 70 cities and towns across MO currently contain breed restrictions (24). However, since 2018, at least a dozen municipalities in MO, including Springfield, Liberty and Independence, have repealed BSL, which follows the trend seen in municipalities across the country in recent years (25). Results from this research can be used to assess the impact these repeals may have on dog bite injuries and inform future decisions regarding BSL repeal.

## Methods

### Data sources

Emergency department patient injury data recorded by MO hospitals from 2010 through 2015 were retrieved from the Missouri Public Health Information Management System’s (MPHIMS) Injury – MICA database (26). These data are the most recent available from the Injury MICA database. International Classification of Diseases-9th Revision-Clinical Modification codes were used to identify ED visits related to dog bites (ICD-9 Code E906) for each year and counts were grouped by the patient zip code. Population estimates for 2010 through 2015 were taken from the American Community Survey (ACS) 1-Year Data and aggregated by Zip Code Tabulation Area (ZCTA), the geographic representations of United States Postal Service zip code service areas (27). After merging the ED visits and population estimates by zip code/ZCTA, annual crude estimates for ED visits per 100,000 residents were calculated by dividing the number of ED visits recorded in each zip code by the estimated population for the zip code and multiplying by 100,000. Average crude rates for dog bite-related ED visits across the five-year period were then calculated for each zip code. As part of the MPHIMS confidentiality and data suppression rules, Injury MICA data are suppressed when cell counts of ED visits by year fall below three cases (26). In areas of the state with small populations, rates for dog bites often fell below three per year. To account for the higher likelihood of missing data in areas of the state with fewer residents, analyses were limited to zip codes in the highest quartile of the state’s population (an average above 14,000 residents over the five-year period).

**Figure 1:**
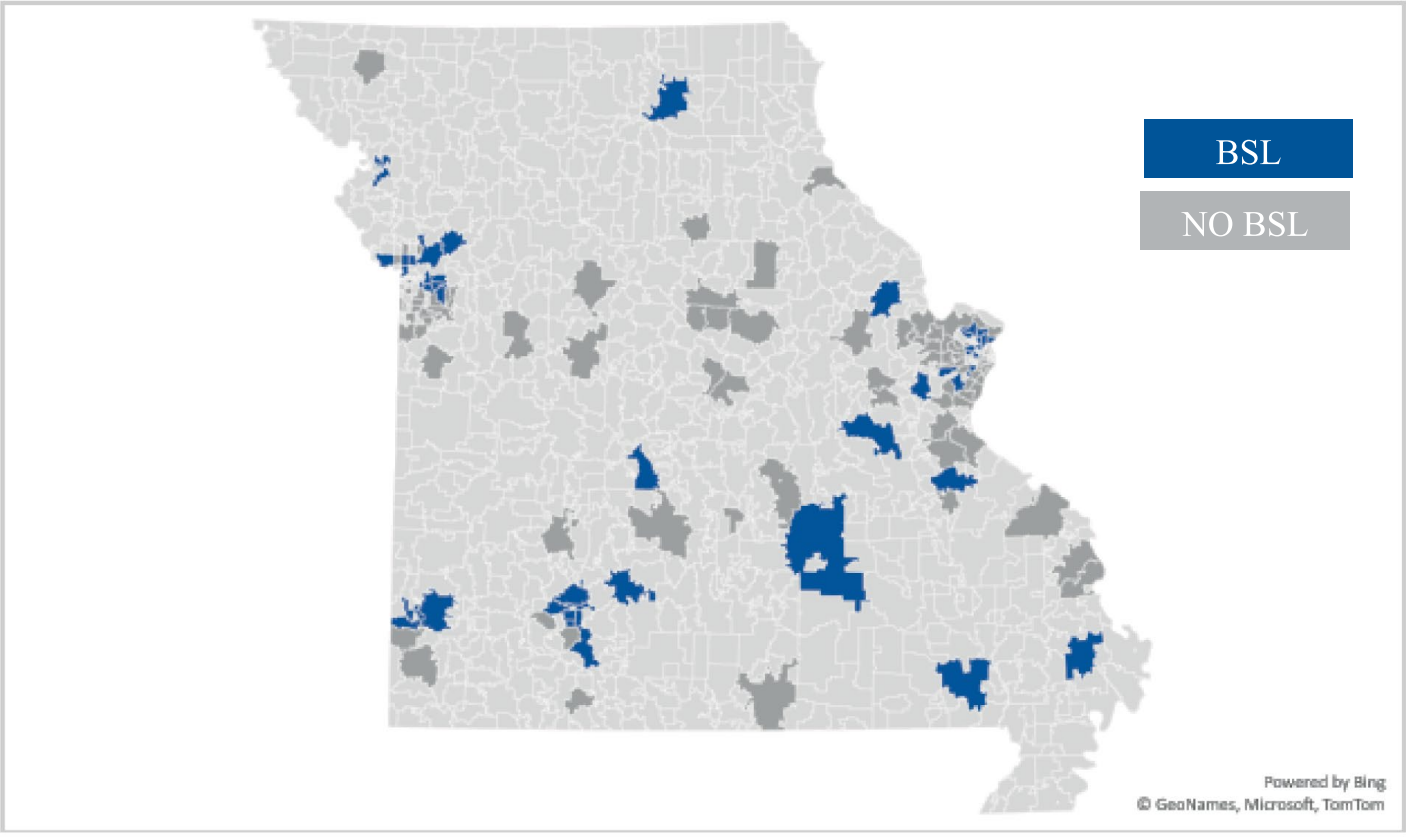
Zip codes included in analytic dataset and BSL status between 2010 and 2015.

Municipalities in MO with BSL during the 2010–2015 period were identified though the online directory bslcensus.com (24). For each municipality listed, current municipal codes were reviewed to confirm that BSL had been enacted and to determine whether the ordinance had been repealed. All zip codes that fell within the municipalities with BSL ordinances (including bans or restrictions) in effect between 2010 and 2015 were indexed and merged with the dog bite-related ED records. The resulting dataset used for the analysis included 142 zip codes, 48 zip codes with BSL and 94 without BSL (Figure 1).

Population and housing estimates used to balance the sample were acquired from the 2015 American Community Survey 5-Year Data, aggregated by ZCTA and merged with the analytic dataset (28). Data include the total population for the period and the proportion of males aged 5 to 9 and males aged 10 to 14 – the populations for which dog bite ED visits are most prevalent. The proportions of renter occupied housing units were also included, as the space limitations of rental units and landlord pet restrictions may be associated with the size and breeds of dogs owned. Complete ED visit data were not available for all six years of the study period for eight zip codes; therefore, person-years were calculated for each zip code using the number of years for which data were available and the estimate of the population. Dog ownership estimates are not available by zip code/ZCTA for 2010–2015. However, ZCTA aggregated 2022 estimates of household pet ownership are available through the Esri ArcGIS GeoEnrichment service (29). Although zip code-level estimates of the total dog population are not available, these data were used to approximate the relative differences in the percentage of households with one dog and percentage of households with two or more dogs across municipalities included in the analysis under the assumption that general trends in dog ownership rates have remained relatively stable in these municipalities since 2015.

### Analysis

All analyses were conducted using R 4.3.1 (30). Bivariate comparisons of dog bite injury rates, population and housing characteristics and dog ownership rates across groups (with and without BSL) were completed using independent t-tests. Propensity scores for the probability of each municipality being in the BSL group were calculated using logistic regression and estimates of population proportions for males aged 5 to 9, males 10 to 14, renter occupied housing units, households with one dog and households with two or more dogs as covariates. Municipalities were matched using the Full Match Method in the MatchIt R package (31). Standardized mean differences (SMD) and variance ratios (VR) were used to evaluate the balancing of covariates related to selection, with balancing thresholds set to −0.1/+0.1 for SMD and 0.8 and 1.25 for VR (32). Negative binomial regression was used for all predictive models, with the total ED visits for dog bite related injury as the dependent variable and the log person-years included as an offset. Models with BSL predicting injury rate were tested with and without covariates.

## 3 Results

**Figure 2:**
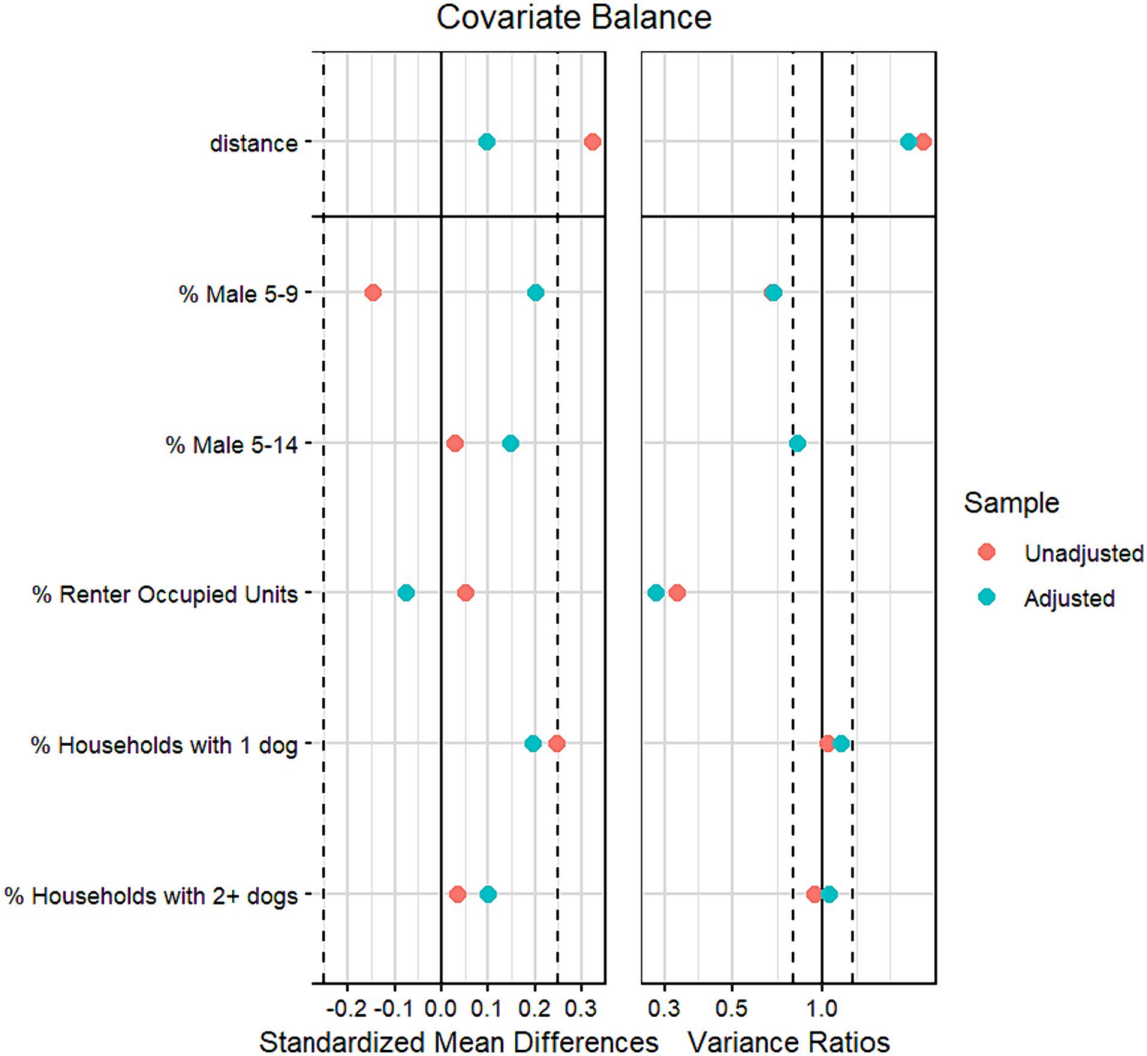
Standardized mean differences and variance ratios for balanced and unbalanced groups.

Differences between BSL and non-BSL municipalities with respect to the average rate of ED visits for a dog bite injury, population and housing estimates and dog ownership estimates are included in Table 1. The overall rate of ED visits for dog bite injuries from 2010 to 2015 averaged 101/100,000 residents. No statistically significant differences in injury rates were found between municipalities with or without BSL, or between population, housing and dog ownership estimates. Accordingly, SMDs and VRs were minimally improved through matching (Figure 2). Notably, while the SMD for the proportion of renter occupied units in each group was relatively balanced, an imbalance in the VR, the result of greater variation in the non-BSL group, remained after matching.

**Table 1:** Characteristics of Missouri municipalities with and without breed-specific legislation.

	Mean (standard deviation)			
	No BSL (*n* = 94)	BSL (*n* = 48)	Total (*n* = 142)	*p*-value^1^
Average crude rate of dog bite-related emergency department visits 2010–2015 (per 100 k residents)	98.93 (33.27)	104.17 (34.94)	100.73 (33.82)	0.39
% males 5 to 9 years old (2011–2015)	3.40 (0.87)	3.24 (0.69)	3.35 (0.81)	0.27
% males 10 to 14 years old (2011–2015)	3.30 (0.93)	3.29 (0.83)	3.30 (0.89)	0.94
% renter occupied units (2011–2015)	30.43 (13.58)	31.14 (8.44)	30.67 (12.06)	0.74
% households with one dog (2022)	24.56 (2.89)	25.28 (2.94)	24.80 (2.92)	0.16
% households with two or more dogs (2022)	15.70 (4.50)	15.85 (4.34)	15.75 (4.43)	0.86

^1^Independent sample *t*-test.

Incidence rate ratios generated from a regression model predicting ED rates without covariates or balancing (1) and a balanced regression model with population, housing and dog ownership covariates (2) are presented in Table 2. Breed-Specific Legislation was not found to be associated with ED visits in either model. After matching the sample on population, housing and dog ownership estimates, the rate ratio of ED visits for dog bite related injuries increased with greater proportions of males aged 5 to 9 in the population. Specifically, with every 1% increase in the population of males aged 5 to 9 the incidence rate ratio is predicted to increase by 13.8% (*p* < 0.01). The proportion of renter occupied units was also found to be positively associated with injury, with a 1.3% increase in incidence rate ratio for every 1% increase in renter occupied units (*p* < 0.01).

**Table 2:** Negative binomial regression results for Breed-Specific Legislation predicting emergency department visits for dog bite injuries.

	Dependent variable: rate of dog bite-related emergency department visits in Missouri 2010–2015 (per person-year)	
	Incidence Rate Ratio (SE)	
	No balancing or covariates (1)	Propensity score matching & covariates (2)
Breed-Specific Legislation (Yes = 1)	1.086 (1.061)	1.076 (1.055)
% males 5 to 9 years old (2011–2015)		1.138*** (1.038)
% males 10 to 14 years old (2011–2015)		1.006 (1.039)
% renter occupied units (2011–2015)		1.013*** (1.003)
% households with one dog (2022)		0.993 (1.014)
% households with two or more dogs (2022)		1.003 (1.009)
Constant	0.001*** (1.035)	0.0005 (1.366)
Log Likelihood	−720.745	−705.995
Theta	9.895*** (1.270)	12.356*** (1.614)
Akaike Inf. Crit.	1,445.490	1,425.991

^***^p < 0.01.

## Discussion

Consistent with previous research (17–22), this study found that the rate of ED visits for dog bite-related injuries was not associated with dog breed restrictions placed on the residents of communities. Estimates of having a single dog or multiple dogs in a home were not associated with rates of dog bite injury. Also consistent with injury epidemiology research, incidence of injury from a dog bite that prompted an ED visit was found to be greatest among male children between the ages of 5 and 9 years-old, regardless of how many dogs were in the community or what breed restrictions were placed on dog owners. Space limitations may inherently influence the number, size and breed of dogs owned by renters and landlord-imposed pet restrictions are widespread, systematically limiting dog ownership among renters (33). While BSL restricts ownership based on breed, landlord-imposed bans are less specific and restrict any type of dog the landlord considers to be more volatile or unwelcome. Yet, the rate of dog bite injury was found to *increase* with the proportion of renters in a community. Results of this study suggest that the approach of both landlords and government agencies to use ownership restrictions as a means of mitigating risk is fundamentally unsound.

The 2010–2015 rate of dog bite-related ED visits for the sample of MO communities included in this study was 101 visits per 100,000 residents, while the national average over that period was 112 visits per 100,000. There is insufficient evidence to demonstrate Missouri residents had an outsized risk of injury from a dog bite, yet with over 70 BSL ordinances throughout the state, the local response to the perceived threat of dog bites from specific breeds was quite substantial. Injury rates in communities with BSL included in this study were 104 per 100,000 residents. While it is impossible to know what this rate would be if BSL were not enacted in these communities, results from this study suggest that a school-aged male child is just as likely to end up in the ED for a dog bite in a city with a breed restriction than one without and it appears to be unrelated to the number of dogs in the community. There is no benefit to parents of these children that the breed of dog that caused the injury was or was not permitted within city limits. Absent any data demonstrating that a breed restriction decreases the overall rate of dog bite injury, there is insufficient evidence to use this strategy to promote public health and safety.

Although select population and household variables were found to be associated with dog bite injuries, many other factors not considered in this analysis may be associated with dog bite injury risk. Renter occupied housing units were included in the model as a potential proxy for dog breed and size. However, rental units may also be associated with sociodemographic or environmental variables. For example, *per capita* income and education, which were found to be associated with dog bite injury in New York City (7), or housing vacancy rates, which were found to be associated with dog bite injury in Detroit (34). Coupling this with the strong relationship between dog bite injury and household composition (particularly, the presence of young children), public health and safety planning that focuses on the needs of parents and the responsibilities of both parents and dog owners would likely be more effective at preventing injury than strategies focused on the physical characteristics of the dogs they own.

While the data included in this study provided meaningful metrics to assess policies restricting dog breeds, more detailed information, such as the breeds of dogs involved in injuries, information about the ED patients and the overall incidence of dog bites in a community, would strengthen the validity of the results. Unfortunately, these data are scarce and, even if available, infamously unreliable. The lack of ED data from less-populated areas also limited our ability to assess BSL statewide. The quasi-experimental method of propensity score matching allowed for less biased causal inferences, similar to analyses of data from randomized groups. However, matching only accounted for factors known to be associated with the predictor or outcome variables. Unknown or unavailable explanatory variables could not be accounted for, which are assumed to be proportionally distributed through true randomization. Nonetheless, matching provided more context to the analyses, such that alternative explanations for the relationships between variables were limited.

## Conclusion

We have found that policies in MO that restrict dog ownership based on breed have not reduced the risk of ED visits for injury from dog bites, and no relationship was found between estimates of dog ownership and rates of injury. As such, there appears to be no greater risk to public safety as dog ownership continues to increase in popularity and local and state governments move to repeal existing breed bans. Consistent with previous research, rates of injury were, however, associated with population and household characteristics, highlighting the need to shift the focus of injury prevention to factors associated with the owner.

## Data availability statement

The original contributions presented in the study are included in the article/supplementary material, further inquiries can be directed to the corresponding author.

## Ethics statement

Ethical approval was not required for the study involving humans in accordance with the local legislation and institutional requirements. Written informed consent to participate in this study was not required from the participants or the participants’ legal guardians/next of kin in accordance with the national legislation and the institutional requirements.

## Author contributions

BW: Conceptualization, Data curation, Formal analysis, Methodology, Visualization, Writing – original draft, Writing – review & editing. MG: Conceptualization, Investigation, Methodology, Supervision, Writing – review & editing.

## References

[1] American Pet Products Association. APPA national pet owners survey 2015-2016. Greenwich, CT: American Pet Products Association. (2015).

[2] American Pet Products Association. APPA national pet owners survey 2021-2022. (2021).

[3] Centers for Disease Control and Prevention. National Center for Injury Prevention and Control. Web-based injury statistics query and reporting system (WIAQARS). (2023). Available at: https://wisqars.cdc.gov/ (Accessed June 8, 2023).

[4] Weiss HB Friedman DI Coben JH. Incidence of dog bite injuries treated in emergency departments. JAMA. (1998) 279:51–3. doi: 10.1001/jama.279.1.51 9424044

[5] Rhea SK Weber DJ Poole C Waller AE Ising AI Williams C. Use of statewide emergency department surveillance data to assess incidence of animal bite injuries among humans in North Carolina. J Am Vet Med Assoc. (2014) 244:597–603. doi: 10.2460/javma.244.5.597, PMID: 24548236

[6] Loder RT. The demographics of dog bites in the United States. Heliyon. (2019) 5:e01360. doi: 10.1016/j.heliyon.2019.e01360, PMID: 30957043 PMC6431755

[7] Tuckel PS Milczarski W. The changing epidemiology of dog bite injuries in the United States, 2005–2018. Inj Epidemiol. (2020) 7:1. doi: 10.1186/s40621-020-00281-y 33129353 PMC7603431

[8] Campagna RA Roberts E Porco A Fritz CL. Clinical and epidemiologic features of persons accessing emergency departments for dog and cat bite injuries in California (2005–2019). J Am Vet Med Assoc. (2023) 261:723–32. doi: 10.2460/javma.22.11.0494, PMID: 36853875

[9] Morrill K Hekman J Li X McClure J Logan B Goodman L. Ancestry-inclusive dog genomics challenges popular breed stereotypes. Science. (2022) 376:eabk0639. doi: 10.1126/science.abk0639 35482869 PMC9675396

[10] Patronek GJ Sacks JJ Delise KM Cleary DV Marder AR. Co-occurrence of potentially preventable factors in 256 dog bite–related fatalities in the United States (2000–2009). J Am Vet Med Assoc. (2013) 243:1726–36. doi: 10.2460/javma.243.12.1726, PMID: 24299544

[11] Ott SA Schalke E von Gaertner AM Hackbarth H. Is there a difference? Comparison of golden retrievers and dogs affected by breed-specific legislation regarding aggressive behavior. J Vet Behav. (2008) 3:134–40. doi: 10.1016/j.jveb.2007.09.009

[12] Kim SE Arzi B Garcia TC Verstraete FJ. Bite forces and their measurement in dogs and cats. Front Vet Sci. (2018) 5:76. doi: 10.3389/fvets.2018.00076, PMID: 29755988 PMC5932386

[13] Creedon N Ó Súilleabháin PS. Dog bite injuries to humans and the use of breed-specific legislation: a comparison of bites from legislated and non-legislated dog breeds. Ir Vet J. (2017) 70:23. doi: 10.1186/s13620-017-0101-1 28736610 PMC5521144

[14] Ellis JL Thomason J Kebreab E Zubair K France J. Cranial dimensions and forces of biting in the domestic dog. J Anat. (2009) 214:362–73. doi: 10.1111/j.1469-7580.2008.01042.x, PMID: 19245503 PMC2673787

[15] Patronek GJ Slater M Marder A. Use of a number-needed-to-ban calculation to illustrate limitations of breed-specific legislation in decreasing the risk of dog bite–related injury. J Am Vet Med Assoc. (2010) 237:788–92. doi: 10.2460/javma.237.7.788, PMID: 20919843

[16] Cook RJ Sackett DL. The number needed to treat: a clinically useful measure of treatment effect. BMJ. (1995) 310:452–4. doi: 10.1136/bmj.310.6977.452, PMID: 7873954 PMC2548824

[17] Clarke NM Fraser D. Animal control measures and their relationship to the reported incidence of dog bites in urban Canadian municipalities. Can Vet J. (2013) 54:145–9. PMID: 23904637 PMC3552590

[18] Mora E Fonseca GM Navarro P Castaño A Lucena J. Fatal dog attacks in Spain under a breed-specific legislation: a ten-year retrospective study. J Vet Behav. (2018) 25:76–84. doi: 10.1016/j.jveb.2018.03.011

[19] Súilleabháin PÓ. Human hospitalisations due to dog bites in Ireland (1998–2013): implications for current breed specific legislation. Vet J. (2015) 204:357–9. doi: 10.1016/j.tvjl.2015.04.021, PMID: 25957919

[20] Klaassen B Buckley JR Esmail A. Does the dangerous dogs act protect against animal attacks: a prospective study of mammalian bites in the accident and emergency department. Injury. (1996) 27:89–91. doi: 10.1016/0020-1383(96)83411-5, PMID: 8730379

[21] Cornelissen JM Hopster H. Dog bites in the Netherlands: a study of victims, injuries, circumstances and aggressors to support evaluation of breed specific legislation. Vet J. (2010) 186:292–8. doi: 10.1016/j.tvjl.2009.10.001, PMID: 19879172

[22] Nilson F Damsager J Lauritsen J Bonander C. The effect of breed-specific dog legislation on hospital treated dog bites in Odense, Denmark—a time series intervention study. PLoS One. (2018) 13:e0208393. doi: 10.1371/journal.pone.0208393, PMID: 30586418 PMC6306151

[23] Benedetto U Head SJ Angelini GD Blackstone EH. Statistical primer: propensity score matching and its alternatives. Eur J Cardiothorac Surg. (2018) 53:1112–7. doi: 10.1093/ejcts/ezy167, PMID: 29684154

[24] BSL Census. List of breed specific legislation in Missouri. (2023). Available at: https://bslcensus.com/bsl/missouri/ (Accessed June 8, 2023).

[25] Pitbull Info. BSL continues to crumble. (2023). Available at: https://www.pitbullinfo.org/bsl-continues-to-crumble.html (Accessed June 8, 2023).

[26] Missouri Department of Health and Senior Services. Missouri public health information management system. (2023). Available at: https://healthapps.dhss.mo.gov/MoPhims/QueryBuilder?qbc=IM&q=1&m=1 (Accessed July 8, 2022).

[27] United States Census Bureau. American community survey 1-year data (2005-2021). (2022). Available at: https://www.census.gov/data/developers/data-sets/acs-1year.html (Accessed September 21, 2022).

[28] US Census Bureau. American community survey 5-year data (2011–2015). (2016). Available at: https://www.census.gov/programs-surveys/acs/technical-documentation/table-and-geography-changes/2015/5-year.html (Accessed September 21, 2022).

[29] Esri. GeoEnrichment service (2023). Available at: https://developers.arcgis.com/documentation/mapping-apis-and-services/demographics/services/geoenrichment-service/

[30] R Core Team. R: a language and environment for statistical computing. Vienna, Austria: R Foundation for Statistical Computing (2016).

[31] Ho D Imai K King G Stuart E Whitworth A Greifer N. MatchIt: nonparametric preprocessing for parametric causal inference. (2023). Available at: https://cran.r-project.org/web/packages/MatchIt/index.html

[32] Zhang Z Kim HJ Lonjon G Zhu Y. Balance diagnostics after propensity score matching. Ann Trans Med. (2019) 7:16. doi: 10.21037/atm.2018.12.10 PMC6351359 30788363

[33] O’Reilly-Jones K. When fido is family: how landlord-imposed pet bans restrict access to housing. Columbia J Law Soc Problems. (2018) 52:427.

[34] Reese LA Vertalka JJ Wilkins MJ Pizarro JM. Demographic and urban environmental variables associated with dog bites in Detroit. J Am Vet Med Assoc. (2019) 254:986–90. doi: 10.2460/javma.254.8.986, PMID: 30938618

